# Increased inflammatory response is associated with less favorable functional results 5 years after total knee arthroplasty

**DOI:** 10.1007/s00167-021-06836-w

**Published:** 2022-02-11

**Authors:** Jörg Lützner, Franziska Beyer, Cornelia Lützner, Peter Thomas, Burkhard Summer

**Affiliations:** 1 University Center of Orthopaedic, Trauma and Plastic Surgery, University Hospital, TU Dresden, Fetscherst. 74, 01307 Dresden, Germany; 2 Department of Dermatology und Allergology, Ludwig-Maximilias-University, Frauenlobstraße 9–11, 80337 Munich, Germany

**Keywords:** Total knee arthroplasty, Total knee replacement, Coating, Allergy, Hypersensibility, Cytokines, Results, Patient-reported outcome

## Abstract

**Purpose:**

Allergy against implant materials is discussed controversially and still not fully understood. Despite these controversies, a relevant number of patients receive hypoallergenic knee implants. The aim of this study was to compare a new coating system with the standard implant in total knee arthroplasty (TKA). Additionally, the influence of proinflammatory cytokines on patient-reported outcome measures (PROMs) was investigated.

**Methods:**

120 patients without known metal allergy and without previous metal implants were included. The patients were randomized to receive a coated or standard TKA of the same knee system. 105 patients completed the 5 year follow-up. Patient-reported outcome measures (PROMs) including knee function (Oxford Knee Score, OKS), quality of life (SF36) and UCLA activity scale were assessed. Additionally, several cytokines with a possible role in implant allergy were measured in patient`s serum (IL-1beta, IL-5, IL-6, IL-8, IL-10, IP-10, IFN γ, TNF α). Group comparison was performed using Mann–Whitney *U* test for continuous values and chi-square test for categorical values.

**Results:**

There were no differences in PROMs between both groups at any follow-up. The majority of patients demonstrated no elevation of the measured blood cytokines. The blood cytokine pattern after 5 years demonstrated no differences between study groups. There was a significant association between elevated IL-8 values and worse results in the overall OKS (*p* = 0.041), the OKS function component (*p* = 0.004), the UCLA activity scale (*p* = 0.007) and the physical component of SF36 (*p* = 0.001).

**Conclusion:**

There were no problems with the new coating during mid-term follow-up and no differences in PROMs between coated and standard TKA. Patients with an increased inflammatory response demonstrated worse functional results, regardless of the implant.

**Level of evidence:**

I.

**Clinical trial registration:**

The study protocol was registered in the US National Institutes of Health’s database (http://www.clinicaltrials.gov) registry under NCT00862511.

## Introduction

There is still a debate if allergies against implant materials exist or not, which diagnostic test may be used and if hypoallergenic implants may be advantageous in these patients [1, 11, 16, 17]. Despite these controversies, the prevalence of contact allergies against implant materials is high [21] and affected patients usually ask for hypoallergenic implants. These hypoallergenic implants are usually coated or ceramised standard implants. These surface modifications are supposed to reduce the release of metal ions and metallo-organic complexes in an attempt to prevent adverse biological reactions in the knee joint. Additionally, it has been demonstrated that these surface modifications result in less polyethylene wear in vitro. In the German Arthroplasty Registry (EPRD), 8.6% of all TKA and 11.5% of all unicondylar knee arthroplasties (UKA) in the year 2019 were hypoallergenic implants [4]. Studies have demonstrated that some of these coatings in UKA and TKA are safe and resulted in similar outcomes compared to standard implants during short and mid-term follow-up [2, 3, 18]. Unfortunately, there have been reports about less favourable results with such hypoallergenic materials [6, 12, 25]. In a retrieval study, 21% of the TKA demonstrated coating delaminations which might affect the performance of the coating [6, 12, 25]. To avoid delamination and improve the mechanical characteristics of the implant surface, a seven-layer coating system (AS—Advanced Surface, BBraun Aesculap, Tuttlingen, Germany) had been developed. The coating consists of seven layers resulting in a gradient change in stiffness between the implant body and the final coating layer [20].

It is known that an inflammatory response occurs after a surgery, especially if a large metal implant is used. A variety of mediators has been reported to play a role in this inflammatory response, such as Interleukin 1beta (IL-1beta), IL-6, IL-17A, Interferon gamma (IFN γ) or Tumor necrosis factor α (TNF α) [22]. Thomas et. al. [24] have demonstrated that pro-inflammatory IL-8 and anti-inflammatory IL-10 were elevated 5 years after surgery in standard TKA compared to coated TKA. There were, however, no differences in clinical outcomes. Therefore, the significance of these cytokine changes remains unknown.

This study was initiated to investigate the AS coating system in comparison to the standard implant. The hypothesis was that the new coating system would demonstrate similar results to the standard TKA. This is a mid-term follow-up report of an earlier published study [15], comparing the mid-term outcomes between coated and uncoated TKA. Additionally, the influence of proinflammatory cytokines on patient-reported outcome measures (PROMs) was investigated.

## Materials and methods

After institutional review board approval (EK 322122008) and registration at ClinicalTrials.gov (NCT00862511) a randomized-controlled trial was performed, which has been described before [14, 15]. Patients with end-stage osteoarthritis of the knee which were scheduled for an unconstraint TKA without known hypersensitivities against implant materials and without any metal implant were eligible. All patients underwent patch testing and four patients revealed a previously unknown hypersensitivity against implant materials. According to local guidelines, all four patients received a coated implant, although two were randomized to receive a standard implant. Patients with the need for a higher constraint TKA were excluded. After informed consent, a total of 120 patients received according to the randomization list a standard or coated cruciate-retaining TKA without patellar resurfacing (Columbus, BBraun Aesculap, Tuttlingen, Germany). Both implants consisted of a CoCrMo-alloy (ISO 5832-4). The coated TKA had an additional multilayer coating system (Advanced Surface, AS) which was applied on the CoCrMo knee implants using a physical vapour deposition (PVD) method with a total thickness of about 4 µm [20]. All surgeries were performed by two experienced TKA surgeons using a medial parapatellar approach and a measured resection technique. All implants were cemented.

**Fig. 1:**
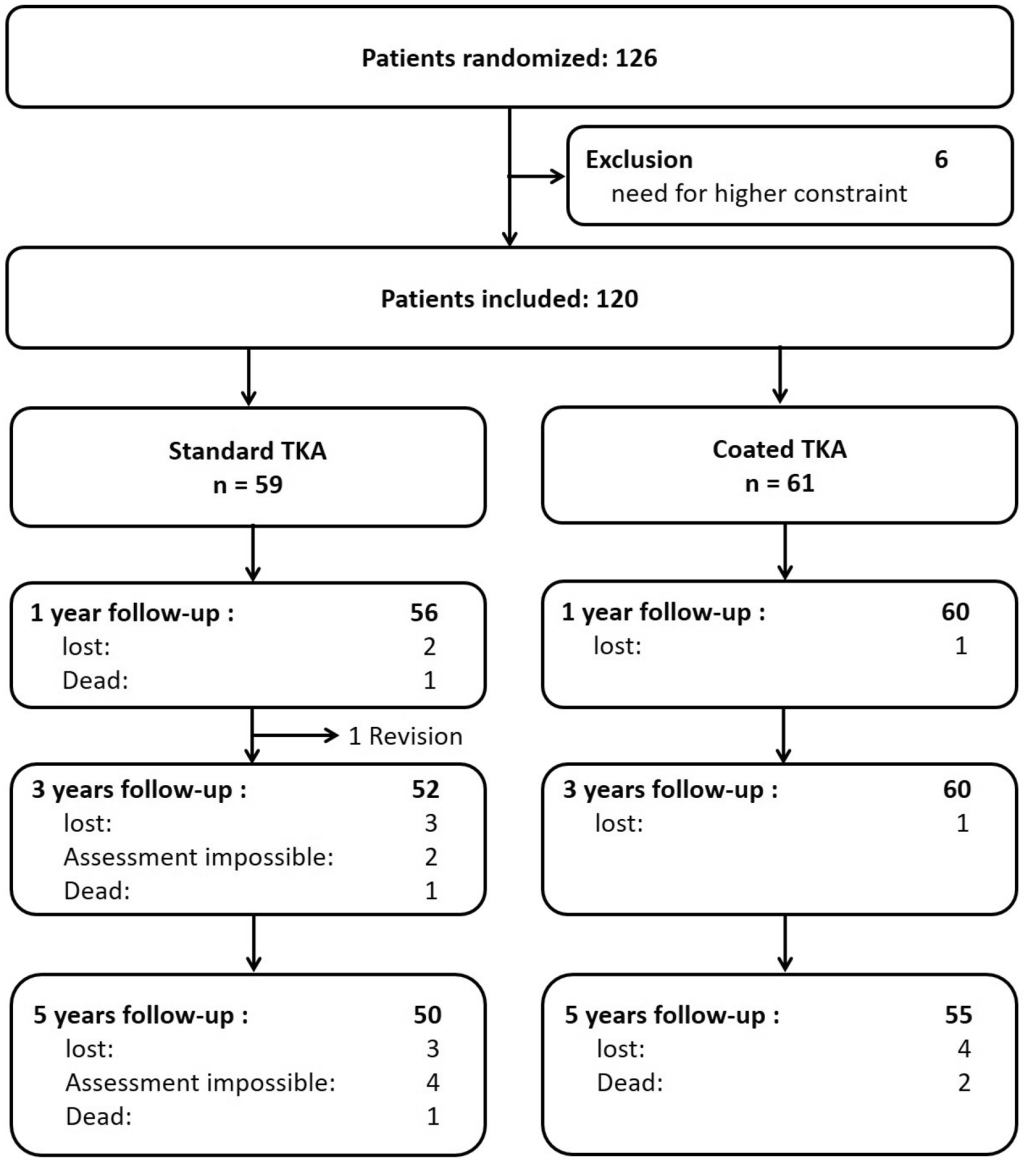
Flowchart of study patients

Patients were seen by a study nurse before, 3 months, 1 year, 3 and 5 years after surgery. Knee Function (Oxford Knee Score, OKS, Knee Society Score, KSS), physical activity (University of Los Angeles (UCLA) activity scale) and health-related quality of life (SF36) were obtained. Furthermore, patients were asked about their overall satisfaction with the outcome of the TKA on a visual analogue scale (VAS) from 0 (not satisfied) to 10 (very satisfied). 105 patients completed the five-year follow-up (Fig. 1).

Until 5 year follow-up, 19 patients in the coated and 9 patients in the standard group received a TKA at the contralateral knee. In all patients, the same implant as at the index knee was used. Additionally, 5 patients received a total hip arthroplasty.

### Blood cytokine pattern

At the 5 year follow-up, valid blood cytokine pattern were available from 92 patients from cryopreserved serum samples, which were stored at − 20 °C. The blinded samples were assessed for the presence and concentration of 10 cytokines by a multiplex cytometric bead assay (CBA; BD Biosciences, Heidelberg, Germany) via flow cytometry using a FACS canto (BD Biosciences, Heidelberg, Germany). The panel of cytokines included—with the restriction of sometimes overlapping functions—inflammatory [IL-1beta, IL-5, IL-6, IFN γ, TNF α), chemoattractant (IL-8, Interferon gamma-induced protein 10 (IP-10)] and immune regulating (IL-10) factors. The respective detection limits were < 0.01 pg/ml. The results were additionally evaluated by double assessment of 10 randomly selected blood samples.

### Statistical analysis

Data description was based on median (25th; 75th percentile) for continuous values and absolute and relative frequencies for categorical values. Comparisons between treatment groups were done by Mann–Whitney *U* test for continuous values and chi-square test for categorical values. Differences between normal and elevated cytokine levels with regard to PROMs were also analyzed by Mann–Whitney *U* test. Significance level was set at *p* < 0.05. The software SPSS (release 26 for Windows) was used for data analysis. Sample size calculation was initially performed for detection of a difference in metal ion levels of 0.67 μg/l. With current sample size a difference of 3 points in the Oxford Knee Score could be detected which is below the minimally clinical relevant difference.

## Results

Until the 5 year follow-up, there was one revision due to patellar instability, three patients died, seven were lost to follow-up and in four patients assessment was not possible due to other serious diseases. 105 patients completed the 5 year follow-up, 50 in the standard group and 55 in the coated group. The two groups were not different with regard to preoperative and perioperative data, such as gender, age, body mass index (BMI), operative time and co-morbidities (Table 1).

**Table 1:** Characteristics of patients with 5 year follow-up (median (25th; 75th percentile) or absolute (relative) frequency)

	Standard TKA (*n* = 50)	Coated TKA (*n* = 55)	*p* value
Age at surgery [years]	70 (63; 73)	66 (58; 74)	n.s
Female gender	28 (56.0%)	29 (52.7%)	n.s
Comorbidities			
ASA grade 1 or 2	31 (62.0%)	35 (63.6%)	n.s
ASA grade 3 or 4	19 (38.0%)	20 (36.4%)	
BMI [kg/m^2^]	29.8 (27.0; 31.8)	30.3 (28.1; 33.5)	n.s
Cut sew time [minutes]	87.5 (73; 95)	86 (75; 100)	n.s

There were no differences in PROMs between both groups at any follow-up (Table 2).

**Table 2:** PROMs of patients with 5 year follow-up (median (25th; 75th percentile) or absolute (relative) frequency)

	Standard TKA (*n* = 50)	Coated TKA (*n* = 55)	*p* value
Oxford Knee Score [0–48]			
Before surgery	23 (16; 28)	21 (18; 27)	n.s
5 year follow-up	41.5 (36; 44)	39 (33; 44)	n.s
OKS pain component subscale [0–100]			
Before surgery	46.4 (28.6; 57.1)	42.9 (28.6; 53.6)	n.s
5 year follow-up	92.9 (78.6; 96.4)	85.7 (71.4; 96.4)	n.s
OKS function component subscale [0–100]			
Before surgery	50 (35; 60)	50 (35; 60)	n.s
5 year follow-up	80 (65; 85)	75 (60; 85)	n.s
Knee society knee score [0–100]			
Before surgery	48.5 (31; 59)	46 (38; 54)	n.s
5 year follow-up	91 (75; 95)	91 (72; 99)	n.s
Knee society function score [0–100]			
Before surgery	50 (40; 60)	50 (45; 60)	n.s
5 year follow-up	70 (60; 80)	70 (60; 90)	n.s
Satisfaction with TKA			
VAS (minimum 0, maximum 10)	8.5 (7; 9.5)	8.5 (7; 9.5)	n.s
Health-related quality of life (Short-form 36)			
Physical summary scale			
Before surgery	29.4 (22.1; 34.3)	27.2 (22.3; 32.8)	n.s
5 year follow-up	44.1 (38.1; 50.3)	42.7 (32.1; 55.1)	n.s
Mental summary scale			
Before surgery	55.1 (41.3; 65.3)	54.3 (42.5; 60.7)	n.s
5 year follow-up	54.8 (48.5; 60.1)	52 (41.2; 57.2)	n.s

The blood cytokine pattern after 5 years demonstrated no differences between study groups (Table 3). The majority of patients demonstrated no elevation of the measured blood cytokines. Few patients had measurable levels of IL-8 (*n* = 14, 15%), IL-6 (*n* = 1, 1%), IFN γ (*n* = 5, 5%) and TNF α (*n* = 1, 1%). IP-10 was present in nearly all patients (median 31.0 pg/ml in standard TKA, 33.4 pg/ml in coated TKA).

**Table 3:** Blood cytokine levels in standard and coated TKA (absolute and relative frequencies of values above 0.01 pg/ml)

	Standard TKA		Coated TKA		*p* value
	*n*	%	*n*	%	
IP-10	44	93.6	43	95.6	n.s
IL-1beta	0		0		
IL-4	0		0		
IL-5	0		0		
IL-6	1	2.1	0	0.0	n.s
IL-8	6	12.8	8	17.8	n.s
IL-10	0		0		
IFN γ	2	4.3	3	6.7	n.s
TNF α	1	2.1	0		n.s

There was a significant association between elevated IL-8 values and worse results in the overall OKS (*p* = 0.041), the OKS function component (*p* = 0.004), the UCLA activity scale (*p* = 0.007) and the physical component of SF36 (*p* = 0.001), see Table 4 for details.

**Table 4:** Association of PROMs and inflammatory response at the 5 year follow-up (median (25th; 75th percentile) or absolute (relative) frequency)

	IL 8 = 0 pg/ml (*n* = 78)	IL 8 > 0.01 pg/ml (*n* = 14)	*p* value
Oxford knee score [0–48]			
Before surgery	23 (18; 28)	21 (14; 27)	n.s
5 year follow-up	42 (36; 44)	36 (33; 42)	0.041
OKS pain component subscale [0–100]			
Before surgery	46.4 (28.6; 53.6)	39.3 (25; 60.7)	n.s
5 year follow-up	91.1 (78.6; 96.4)	87.5 (75; 96.4)	n.s
OKS function component subscale [0–100]			
Before surgery	52.5 (40; 60)	40 (35; 50)	n.s
5 year follow-up	80 (70; 90)	65 (55; 70)	0.004
Knee society knee score [0–100]			
Before surgery	49 (34; 59)	46 (41; 55)	n.s
5 year follow-up	91 (74; 98)	90.5 (75; 95)	n.s
Knee society function score [0–100]			
Before surgery	50 (50; 60)	47.5 (40; 60)	n.s
5 year follow-up	70 (60; 90)	60 (40; 70)	n.s
Satisfaction with TKA			
VAS (minimum 0, maximum 10)	8.8 (7.5; 9.5)	8.8 (5.5; 10)	n.s
Health-related quality of life (Short-form 36)			
Physical summary scale			
Before surgery	28.2 (23.1; 34.3)	22.3 (20.6; 32.9)	n.s
5 year follow-up	46.5 (38.3; 54.1)	34.9 (29.9; 44.9)	0.005
Mental summary scale			
Before surgery	55.1 (41.8; 63.5)	54.4 (48.6; 60.3)	n.s
5 year follow-up	53.7 (42.3; 58.6)	53.7 (49.1; 59.9)	n.s

## Discussion

The most important finding of this study is the association between increased inflammatory response and worse outcome after TKA. There were, however, no differences between coated and standard TKA implants during mid-term follow-up.

The correlation between hypersensitivities diagnosed by epicutaneous skin testing and deep-tissue hypersensitivities is still a matter of debate and the need of hypoallergenic implants in these patients is discussed controversially [16]. Despite these debates, some patients will need hypoallergenic implants, either because of guidelines to avoid litigation or because they personally demand them. Although better wear resistance in vitro has been proven [20], these hypoallergenic implants demonstrated even higher overall revision rates in the German arthroplasty registry [10]. In this real-world data, the main differences between coated and standard implant groups were the higher rate of metal allergies in female patients in the coated TKA group. It has been suggested that anxiety is the main reason for less favourable results in patients with allergies [5]. However, the increased early revision rate in coated TKA cannot be explained completely by this fact. Because coated and standard TKA are used in different patient populations, a direct comparison is difficult. The performance of a coated implant should therefore be tested in similar patient populations. It is difficult to perform such a study in patients with allergies against implant materials as some guidelines recommend hypoallergenic implants in these patients. Although this is not the population for which hypoallergenic implants were designed, investigation of its performance in patients without allergies against implant materials is reasonable. There are only few studies comparing coated and standard implants [2, 3, 7, 8, 18, 19, 23, 25]. All of them demonstrated similar results, which are consistent to the results of the present study.

To our knowledge, there is only one cohort study [24] comparing cytokine pattern in coated and standard TKA 5 years after surgery. In this study, IL-8 and IL-10 were significantly elevated in the standard TKA group and not elevated in the coated TKA group. Clinical outcome, however, was similar between both groups. These results are in contrast to the present study, in which no difference between both groups could be detected. Not a single patient demonstrated elevated levels of IL-10 and only 15% had elevated IL-8 serum levels. The reasons for the different results remain unclear. The study of Thomas et al. [24] has been performed in three different hospitals and only one hospital used the coated implant. There might be regional differences caused by environmental influences. This possible bias can be avoided in a randomized setting as in the present study. Furthermore, the applied inclusion criteria excluded patients with any other kind of metal implants, which might influence cytokine levels.

In the present study, the overall immune response at 5 years after surgery represented by different cytokines was low. There was no IL-4 and IL-5 production in the patients, which was expected as both cytokines are typical messengers for type 1 allergic reactions (immediate reaction, e.g. hay fever, allergic rhinitis). Similarly, IL-1beta as a proinflammatory cytokine was not measured in the serum of the patients as well as no counter-regulatory IL-10. There was sporadic IL-6 production, which indicates a possible infection [9]. Despite that, there were no other signs for infection in these patients. The two patients from the standard TKA group and the three patients from the coated TKA group with IFN γ production in the blood might have developed a type 4 allergic immune response (delayed type allergic reaction, e.g. contact dermatitis). However, there were no clinical signs at the follow-up visits for a contact dermatitis. IL-8 production was observed in a larger number of patients. This is a rather general proinflammatory cytokine, which has already been observed by other authors in connection with metal exposure. The same applies for IP-10, which is expressed in cells upon exposure to cobalt [13]. These results demonstrate that the immune system deals with the metal implant in the body, but there seem to be additional factors to initiate an increased immune response or allergy.

Interestingly, patients with an increased IL-8 production in the peripheral blood showed lower values in the Oxford Knee Score and in the Physical summary scale of the SF36. The cytokine IL-8 as a proinflammatory cytokine is expressed during inflammatory processes in the body. IL-8 is known as “neutrophil chemotactic factor” which is produced by all cells with Toll-Like-Receptors, e.g. macrophages. The produced IL-8 induces chemotaxis in cells, especially neutrophils and other granulocytes to migrate to the site of the immune reaction. The increased production might be a sign of an immune reaction in which the body has problems to deal with the implant materials, which results in lower mobility and worse knee function.

Limitations include the exclusion of patients with a history of metal hypersensitivity and therefore the target population for coated implants, only four patients with a previously unknown hypersensitivity were included. Two of these patients received another implant than allocated. As explained above, this was inevitable according to local guidelines. Excluding patients with any kind of metal implants was necessary to avoid bias through these implants in different joints. This resulted in a study population with lower musculoskeletal comorbidities, which might have influenced the PROMs. Until final follow-up, a relevant number of patients had TKA surgery at the contralateral knee. Although the same implant as at the index knee was used, this could have influenced cytokine levels. Additionally, cytokine levels before surgery were not available. The study was initially designed to detect differences in metal ion levels. Furthermore, some patients were lost to follow-up resulting in a follow-up rate of 87.5% after 5 years. However, the remaining sample was large enough to detect meaningful differences in PROMs. Finally, the study was not blinded.

## Conclusion

There were no problems with the new coating during mid-term follow-up and no differences in PROMs between coated and standard TKA. The coating can be used safely in patients who need a hypoallergenic implant. Patients with an increased inflammatory response demonstrated worse functional results, regardless of the implant used. This might explain some unsatisfactory results and should be further investigated.

## References

[1] Bravo D, Wagner ER, Larson DR, Davis MP, Pagnano MW, Sierra RJ (2016). No increased risk of knee arthroplasty failure in patients with positive skin patch testing for metal hypersensitivity: a matched cohort study. J Arthroplasty.

[2] D'Ambrosi R, Anghilieri FM, Corona K, Mariani I, Valli F, Ursino N (2021). Similar rates of return to sports and BMI reduction regardless of age, gender and preoperative BMI as seen in matched cohort of hypoallergenic and standard cobalt chromium medial unicompartmental knee arthroplasty. Knee Surg Sports Traumatol Arthrosc.

[3] D'Ambrosi R, Nuara A, Mariani I, Di Feo F, Ursino N, Hirschmann M (2021). Titanium niobium nitride mobile-bearing unicompartmental knee arthroplasty results in good to excellent clinical and radiographic outcomes in metal allergy patients with medial knee osteoarthritis. J Arthroplasty.

[4] Endoprothesenregister Deutschland (EPRD). Jahresbericht 2020. https://www.eprd.de/fileadmin/user_upload/Dateien/Publikationen/Berichte/Jahresbericht2020-Web_2020-12-11_F.pdf 2020. Accessed 07 April 2021

[5] Ferrer T, Hinarejos P, Goicoechea N, Leal-Blanquet J, Sanchez-Soler J, Torres-Claramunt R (2020). Anxiety is the cause of the worse outcomes of allergic patients after total knee arthroplasty. Knee Surg Sports Traumatol Arthrosc.

[6] Galetz MC, Fleischmann EW, Konrad CH, Schuetz A, Glatzel U (2010). Abrasion resistance of oxidized zirconium in comparison with CoCrMo and titanium nitride coatings for artificial knee joints. J Biomed Mater Res B Appl Biomater.

[7] Garrett S, Jacobs N, Yates P, Smith A, Wood D (2010). Differences in metal ion release following cobalt-chromium and oxidized zirconium total knee arthroplasty. Acta Orthop Belg.

[8] Göbel F, Ulbricht S, Hein W, Bernstein A (2008). Radiological mid-term results of total knee arthroplasty with femoral components of different materials. Z Orthop Unfall.

[9] Gollwitzer H, Dombrowski Y, Prodinger PM, Peric M, Summer B, Hapfelmeier A (2013). Antimicrobial peptides and proinflammatory cytokines in periprosthetic joint infection. J Bone Jt Surg.

[10] Grimberg AW, Grupp TM, Elliott J, Melsheimer O, Jansson V, Steinbrück A (2021). Ceramic coating in cemented primary total knee arthroplasty is not associated with decreased risk of revision due to early prosthetic joint infection. J Arthroplasty.

[11] Guenther D, Thomas P, Kendoff D, Omar M, Gehrke T, Haasper C (2016). Allergic reactions in arthroplasty: myth or serious problem?. Int Orthop.

[12] Herbster M, Döring J, Nohava J, Lohmann CH, Halle T, Bertrand J (2020). Retrieval study of commercially available knee implant coatings TiN, TiNbN and ZrN on TiAl6V4 and CoCr28Mo6. J Mech Behav Biomed Mater.

[13] Lawrence H, Deehan D, Holland J, Kirby J, Tyson-Capper A (2014). The immunobiology of cobalt: demonstration of a potential aetiology for inflammatory pseudotumours after metal-on-metal replacement of the hip. Bone Joint J.

[14] Lützner J, Dinnebier G, Hartmann A, Günther KP, Kirschner S (2009). Study rationale and protocol: prospective randomized comparison of metal ion concentrations in the patient's plasma after implantation of coated and uncoated total knee prostheses. BMC Musculoskelet Disord.

[15] Lützner J, Hartmann A, Dinnebier G, Spornraft-Ragaller P, Hamann C, Kirschner S (2013). Metal hypersensitivity and metal ion levels in patients with coated or uncoated total knee arthroplasty: a randomised controlled study. Int Orthop.

[16] Middleton S, Toms A (2016). Allergy in total knee arthroplasty: a review of the facts. Bone Jt J.

[17] Münch HJ, Jacobsen SS, Olesen JT, Menné T, Søballe K, Johansen JD (2015). The association between metal allergy, total knee arthroplasty, and revision: study based on the Danish Knee Arthroplasty Register. Acta Orthop.

[18] Postler A, Beyer F, Lützner C, Tille E, Lützner J (2021). The use of knee prostheses with a hypoallergenic coating is safe in the medium term: a randomized controlled study. Orthopade.

[19] Postler A, Beyer F, Lützner C, Tille E, Lützner J (2018). Similar outcome during short-term follow-up after coated and uncoated total knee arthroplasty: a randomized controlled study. Knee Surg Sports Traumatol Arthrosc.

[20] Reich J, Hovy L, Lindenmaier H-L, Zeller R, Schwiesau J, Thomas P (2010). Preclinical evaluation of coated knee implants for allergic patients. Orthopade.

[21] Schäfer T, Böhler E, Ruhdorfer S, Weigl L, Wessner D, Filipiak B (2001). Epidemiology of contact allergy in adults. Allergy.

[22] Summer B, Paul C, Mazoochian F, Rau C, Thomsen M, Banke I (2010). Nickel (Ni) allergic patients with complications to Ni containing joint replacement show preferential IL-17 type reactivity to Ni. Contact Derm.

[23] Thienpont E (2015). Titanium niobium nitride knee implants are not inferior to chrome cobalt components for primary total knee arthroplasty. Arch Orthop Trauma Surg.

[24] Thomas P, Hisgen P, Kiefer H, Schmerwitz U, Ottersbach A, Albrecht D (2018). Blood cytokine pattern and clinical outcome in knee arthroplasty patients: comparative analysis 5 years after standard versus “hypoallergenic” surface coated prosthesis implantation. Acta Orthop.

[25] van Hove RP, Sierevelt IN, van Royen BJ, Nolte PA (2015). Titanium-nitride coating of orthopaedic implants: a review of the literature. Bio Med Res Int.

